# Hypervirulent *Klebsiella pneumoniae*: Insights into Virulence, Antibiotic Resistance, and Fight Strategies Against a Superbug

**DOI:** 10.3390/ph18050724

**Published:** 2025-05-15

**Authors:** Helal F. Hetta, Fawaz E. Alanazi, Mostafa A. Sayed Ali, Ahmed D. Alatawi, Hashim M. Aljohani, Rehab Ahmed, Nuha A. Alansari, Fahad M. Alkhathami, Alaa Albogmi, Bander M. Alharbi, Hanadi S. Alanzi, Amirah B. Alaqyli, Yasmin N. Ramadan

**Affiliations:** 1 Division of Microbiology, Immunology and Biotechnology, Department of Natural Products and Alternative Medicine, Faculty of Pharmacy, University of Tabuk, Tabuk 71491, Saudi Arabia; rahmed@ut.edu.sa; 2 Department of Pharmacology and Toxicology, Faculty of Pharmacy, University of Tabuk, Tabuk 71491, Saudi Arabia; falanazi@ut.edu.sa; 3 Department of Pharmacy Practice, Faculty of Pharmacy, University of Tabuk, Tabuk 71491, Saudi Arabia; ma-ali@ut.edu.sa; 4 Department of Clinical Pharmacy, College of Pharmacy, Jouf University, Sakaka 72388, Saudi Arabia; adalatawi@ju.edu.sa; 5 Department of Clinical Laboratory Sciences, College of Applied Medical Sciences, Taibah University, Madina 41477, Saudi Arabia; hsnani@taibahu.edu.sa; 6 Department of Pathology and Laboratory Medicine, College of Medicine, University of Cincinnati, Cincinnati, OH 45221, USA; 7 Laboratory Medicine, College of Applied Medical Sciences, Umm Al-Qura University, Jeddah 22231, Saudi Arabia; nalansari@kau.edu.sa; 8 College of Applied Medical Sciences, University of Tabuk, Tabuk 47315, Saudi Arabia; falkathami@ut.edu.sa (F.M.A.); hs.alenzi@ut.edu.sa (H.S.A.); dr.ameerah2001@gmail.com (A.B.A.); 9 Medical Laboratory Technology, King Abdulaziz University, Jeddah 80216, Saudi Arabia; aalbogmi@ut.edu.sa; 10 Medical Laboratory Technology, College of Applied Medical Sciences, University of Tabuk, Tabuk 47524, Saudi Arabia; 411008754@stu.ut.edu.sa; 11 Department of Microbiology and Immunology, Faculty of Pharmacy, Assiut University, Assiut 71515, Egypt; yasmine_mohamed@pharm.aun.edu.eg

**Keywords:** hypervirulent *Klebsiella pneumoniae*, antibiotic resistance, multidrug resistance, pathogenesis, treatment strategies

## Abstract

Community-acquired infections caused by *Klebsiella pneumoniae* (*K. pneumoniae*) have become a significant global health concern, particularly with the emergence of hypervirulent strains (hvKP). These strains are associated with severe infections, such as pyogenic liver abscesses, even in otherwise healthy individuals. Initially reported in Taiwan in the 1980s, hvKP has now spread worldwide. The pathogenicity of hvKP is attributed to an array of virulence factors that enhance its ability to colonize and evade host immune defenses. Additionally, the convergence of hypervirulence with antibiotic resistance has further complicated treatment strategies. As a member of the ESKAPE group of pathogens, *K. pneumoniae* exhibits high resistance to multiple antibiotics, posing a challenge for healthcare settings. This review provides a comprehensive overview of hvKP, highlighting its structural and pathogenic differences from classical *K. pneumoniae* strains, key virulence factors, mechanisms of antibiotic resistance, and the increasing threat of multidrug-resistant hvKP. Lastly, we discuss current treatment guidelines and emerging therapeutic strategies to combat this formidable pathogen.

## 1. Introduction

*Klebsiella pneumoniae* (*K. pneumoniae*) is a gram-negative, encapsulated, non-motile bacterium belonging to the Enterobacteriaceae family. It is an opportunistic pathogen commonly implicated in nosocomial infections, particularly in immunocompromised individuals. *K. pneumoniae* infections cause a wide range of infections, including pneumonia, urinary tract infections, bacteremia, and liver abscesses [1,2,3].

In recent years, the emergence of hvKP has dramatically increased the risk of severe, invasive infections in both immunocompromised and healthy individuals. While cKp primarily affects hospitalized or immunocompromised patients with underlying conditions such as diabetes, malignancy, or recent surgery, hvKp exhibits a unique ability to cause severe, community-acquired infections in otherwise healthy individuals with no known risk factors. This unusual tropism is attributed to the strain’s enhanced virulence arsenal—including hypermucoviscous capsules, siderophore overproduction, and resistance to phagocytosis—which enables it to breach host barriers and evade immune responses more effectively than cKp strains [4,5,6,7,8,9].

Unlike classical *K. pneumoniae* (cKP), which is primarily associated with hospital-acquired infections, hvKP is more often linked to community-acquired infections and exhibits enhanced virulence, leading to a wider spectrum of systemic diseases, including pneumonia, hepatic abscesses, meningitis, and necrotizing fasciitis.

A distinguishing feature of hvKP is its hypermucoviscous phenotype, which was initially considered a defining trait based on the string test (>5 mm viscous string formation) [10]. However, later studies have shown that not all hvKP strains exhibit hypermucoviscousity, while some cKP strains do [11,12,13,14]. This ambiguity necessitates the use of molecular biomarkers for accurate differentiation, including genes such as *iucA*, *rmpA*, *rmpA2*, and *peg-344*, which are associated with hypervirulence [15,16].

The emergence of antibiotic-resistant hvKP strains has exacerbated the threat posed by this pathogen. As part of the ESKAPE group, *K. pneumoniae* demonstrates resistance to multiple antibiotics, including carbapenems and colistin, making treatment increasingly challenging [17]. The convergence of hypervirulence and multidrug resistance in hvKP strains represents a major public health concern, necessitating urgent research into novel therapeutic strategies [16].

This review provides an in-depth analysis of hvKP, examining its structural and pathogenic distinctions from classical *K. pneumoniae*, its mechanisms of antibiotic resistance, and the growing concern of multidrug-resistant hvKP strains. Additionally, we explore current and emerging therapeutic approaches aimed at combating this highly virulent and drug-resistant pathogen.

## 2. Difference Between Classic and Hypervirulent *K. peumoniae*

*Klebsiella pneumoniae* (KP) exists as two major pathotypes: classical KP (cKP) and hypervirulent KP (hvKP), each differing markedly in clinical presentation, host susceptibility, molecular makeup, and virulence mechanisms. Understanding these differences is crucial for improving diagnosis, infection control, and targeted therapy.

Classical KP is predominantly associated with healthcare settings and is considered a typical opportunistic pathogen. It mainly affects immunocompromised or hospitalized patients and is a common cause of hospital-acquired infections, including pneumonia, urinary tract infections, and bloodstream infections [18]. In contrast, hvKP has emerged as a more aggressive variant capable of causing severe community-acquired infections, even in young and otherwise healthy individuals. It is associated with a broader spectrum of invasive diseases, such as pyogenic liver abscess, meningitis, endophthalmitis, and necrotizing fasciitis [11,19,20].

A key phenotypic feature initially used to differentiate hvKP from cKP is hypermucoviscosity. This trait is identified using the “string test”, wherein an inoculation loop forms a viscous string > 5 mm when lifted from a colony, indicating a mucoid phenotype [10,21]. While this test was once considered a hallmark of hvKP, later studies have shown its limitations in specificity. Some classical strains can also demonstrate hypermucoviscosity, and not all hvKP isolates exhibit this phenotype [19,21]. As such, the diagnostic utility of the string test alone is now viewed with caution, prompting the need for more reliable molecular markers.

The true distinction between the two pathotypes lies in their genetic profiles. hvKP carries additional virulence determinants encoded on large plasmids and, in some cases, specific chromosomal loci. Key markers include *rmpA* and *rmpA2*, which enhance capsule production and contribute to the hypermucoviscous phenotype; *iucA* is a gene encoding the aerobactin siderophore system and *peg-344* is a gene linked to enhanced in vivo virulence [21]. These genes are absent in most cKP strains and show a high predictive value (>95% accuracy) for identifying hvKP. A chromosomal version of *rmpA* (*crmpA*) may also support capsule overexpression, further promoting immune evasion.

Capsular serotypes also provide important clues in distinguishing hvKP. Certain capsular types, notably K1, K2, K5, K20, K54, and K57, are predominantly associated with hypervirulent strains [4,22]. Among them, the K1 serotype—frequently linked to sequence type ST23—is most strongly correlated with liver abscesses, while K2 is found across a broader array of sequence types, such as ST65 and ST86. These capsular types confer enhanced resistance to host immune responses, particularly phagocytosis and complement-mediated lysis, giving hvKP a survival advantage in the bloodstream and deep tissues.

A hallmark of hvKP pathogenesis is its superior iron acquisition system, primarily due to elevated siderophore production. Unlike cKP, hvKP produces high levels of aerobactin, a potent siderophore critical for bacterial proliferation in iron-restricted environments such as the human host [23]. This trait significantly contributes to the organism’s virulence and ability to establish infections at diverse anatomical sites.

Moreover, hvKP demonstrates a heightened capacity to evade the immune system. Studies have shown that hvKP strains are more resistant to neutrophil-mediated phagocytosis and complement activation compared to classical strains [24]. This immune resistance facilitates rapid systemic dissemination and increases the risk of metastatic infections—a feature less common in cKP.

Given the limitations of phenotypic tests like the string test, additional functional assays have been proposed. For instance, centrifugation-based mucoviscosity assays assess the ability of bacterial cells to sediment under low-speed centrifugation. hvKP strains often fail to sediment effectively, leaving turbid supernatants, which serve as another indirect measure of hypermucoviscousity [25]. However, molecular genotyping remains the gold standard for reliable differentiation between these pathotypes.

In summary, while both cKP and hvKP belong to the same bacterial species, their clinical behavior, molecular features, and virulence potential are distinct. cKP remains a significant cause of nosocomial infections in vulnerable patients, whereas hvKP poses a growing public health threat due to its capacity to cause life-threatening, community-acquired infections in healthy individuals. Differentiating between the two requires a combination of clinical judgment, phenotypic testing, and molecular diagnostics for accurate identification and effective management.

## 3. Diseases Caused by hvKP

In 1980, cryptogenic hepatic abscess was the first syndrome reported due to hvKP spp. [26,27,28]. The laboratory and clinical manifestations of infection with hvKP spp. are fever, abdominal pain, leukocytosis, and chills [29]. Hepatic abscess due to the infection by the hypervirulent spp. is considered a separate syndrome due to many specific characteristics of the infection [27,30]. Other hepatic abscesses usually occur due to biliary affection or previous interventions but, conversely, hepatic abscess caused by hypervirulent spp. is associated with normal liver function and biliary systems [31].

Usually, the route of entry of the hypervirulent strains is the oropharynx through inhalation, leading to pneumonia. In the 1990s, many reports suggested that *K. pneumoniae* pneumonia acquired from the community comprised about less than 1% of pneumonia cases that needed hospital admission in Argentina, Australia, Europe, and North America [32,33,34,35]. Endophthalmitis is a catastrophic complication to the infection by the hypervirulent spp. and was among the first manifestations distinguishing between cKP and hvKP spp. [26]. Previous endogenous endophthalmitis due to gram-negative bacilli was very rare among healthy individuals until the emergence of the hvKP. However, lately it has been considered as a common complication; about 5% of patients with hypervirulent spp. finally develop endogenous endophthalmitis [36]. On the other hand, exogenous endophthalmitis, which occurs due to trauma or surgery, appears to be very rare and associated with the hypervirulent spp. The typical manifestations of endogenous endophthalmitis due to hvKP are blurred vision of sudden onset, ocular redness, and swelling [37].

In Asia, *K. pneumoniae* has been identified as one of the primary causes of meningitis acquired in the community without head injury or neurosurgery [38,39,40,41]. But recently it has been noticed that the infection spread all over the world with the spread of the hypervirulent spp. [42]. Meningitis caused by the hvKP can be a primary infection or a metastatic one [43,44]. Some cases of hypervirulent klebsiella meningitis presented with multiple and diffuse brain abscesses [45,46]. hvKP may be also associated with ventriculitis [45]. Epidural abscess and subdural empyema were among the manifestations of central nervous system (CNS) infections by the hvKP [11,26,46,47,48,49].

In Taiwan, hvKP has become one of the leading causes of necrotizing fasciitis, with a high risk of mortality [50]. Not only in Southeast Asia, but also all over the world, many cases have been recorded [51,52,53]. Infection is in the form of intramuscular abscesses either alone or with necrotizing fasciitis [27,54]. Also, the infection may result in pyomyositis (more in the lower extremities) either alone or with septic arthritis [37]. Osteomyelitis may also occur and may be multifocal, which may be mistaken as malignancy. The exact diagnosis was made after the progression to necrotizing fasciitis [55].

Deep infections in the neck may be either a primary infection or secondary to metastasis [7,56]. Septic thrombophlebitis may be a complication of abscesses in the neck [57]. The superficial infection of soft tissue and skin may also occur due to disseminated infection by hvKP [58].

Also, the urinary tract can be affected following colon affection ascendingly, but it is very rare [4]. Some patients affected by *K. pneumoniae* show both hepatic abscess and colon cancer at the same time [59].

**Figure 1:**
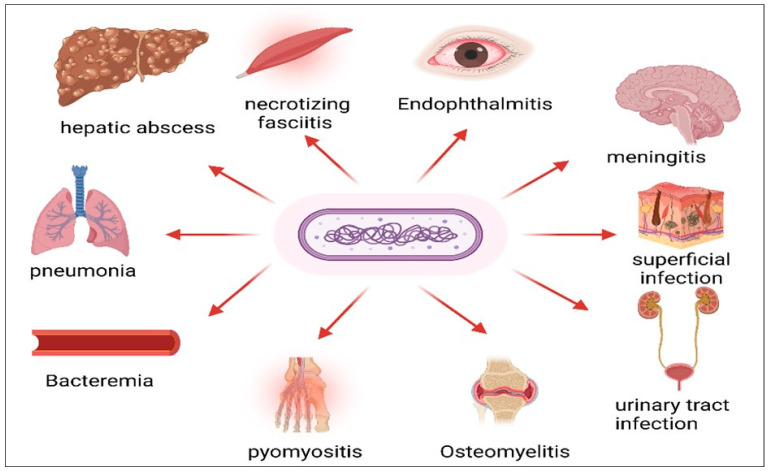
Diseases caused by hvKP. It affects almost all systems and causes endophthalmitis, meningitis, necrotizing fasciitis, pyomylitis, osteomyelitis, pneumonia, bacteremia, hepatic abscess, urinary tract infection, and superficial infection. Created with BioRender.com (https://BioRender.com).

Bacteremia is a very common complication of infection by the hvKP spp. The most common source of bacteremia is pyogenic hepatic abscess [36]. Bacteremia due to hvKP usually has no primary source of infection. Moreover, hypervirulent spp. rarely led to endocarditis. Purulent endocarditis is the most common complication of the hypervirulent spp. [60]; hepatic abscess [61] or a hematogenous route may be the source of infection [46] (Figure 1).

## 4. Discovery and Epidemiology of hvKP

The first recognized report of hvKp was published in 1986 by Liu et al. in Taiwan. They described seven cases of community-acquired *K. pneumoniae* infections presenting with liver abscesses and severe extrahepatic complications, such as prostate abscess, endophthalmitis, and purulent meningitis. Despite receiving aggressive antimicrobial therapy, several patients suffered permanent vision loss, highlighting the pathogen’s virulence [26].

Since then, hvKp has been increasingly reported worldwide, including in countries across Europe, Australia, and North America. Epidemiologically, East and Southeast Asia remain the primary hotspots for hvKp infections [62,63,64]. Taiwan, South Korea, Iran, and China account for a significant proportion of documented cases [8,65]. For instance, a multicenter Chinese study found that 22.8% of invasive *K. pneumoniae* infections were caused by hypervirulent strains [66]. Another study reported that hvKp accounted for 90.9% of pyogenic liver abscesses in that region [52]. In contrast, prevalence rates in Western countries are lower but increasing. In the United States, hvKp has been isolated in approximately 6–8% of clinical *K. pneumoniae* samples, often linked to severe disease and international travel [11].

The continuous emergence of novel hvKp STs—particularly ST23, ST65, and ST86—illustrates the ongoing evolution and adaptation of these strains [67]. In China, multidrug-resistant hvKp (MDR-hvKp) strains are increasingly reported, with ST11 being the most common lineage associated with carbapenem resistance. The global rise of MDR-hvKp poses a dual threat of hypervirulence and antimicrobial resistance, emphasizing the urgency of robust epidemiological surveillance and genomic monitoring [68,69].

In early 2024, the World Health Organization (WHO) reported the global emergence of hvKp ST23 strains harboring carbapenemase genes, indicating resistance to last-line antibiotics. These strains have been detected in at least one country across all six WHO regions, including Algeria, Argentina, Australia, Canada, India, Iran, Japan, Oman, the Philippines, Switzerland, Thailand, the United Kingdom, and the United States. The convergence of hypervirulence and antimicrobial resistance in these strains poses significant challenges for treatment and infection control. The WHO emphasizes the need for enhanced laboratory diagnostic capacities, molecular testing, and surveillance to monitor and mitigate the spread of these high-risk pathogens [70].

## 5. Bacterial Structure

**Figure 2:**
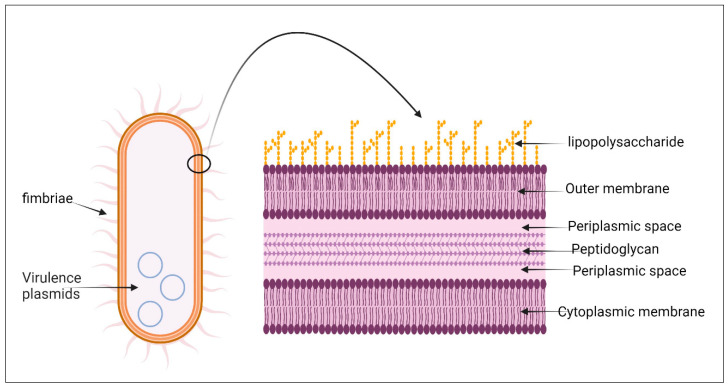
The cell wall structure of *K. pneumoniae*. Like most other gram-negative bacteria, it is composed of an outer membrane that contains lipopolysaccharide, periplasmic spaces, a thin layer of peptidoglycan, and a cytoplasmic membrane. Created with BioRender.com.

Like other members of *Enterobacteriaceae*, hvKP has a lipid bilayer outer membrane consisting of a lipopolysaccharide, lipoprotein, and protein. A capsule of a polysaccharide is present around the outer membrane (Figure 2). Although they are also present in cKP, k1, k2, K5, k20, K54, and k57 are the most prevalent types of the capsule, with k1 along with k2 forming about 70 percent of the hvKP types [4,21,22,71].

But various types of the capsule (for example k47 or k64) are expected to be observed among the virulent group if the classic type continues to acquire the plasmid, which is responsible for its virulence character, as has happened recently [72,73]. The hypervirulent form shows the O-Ag of the lipopolysaccharide. Capsule types of K1 along with K2 are usually present with the O1 O-Ag type, so it is the most prevalent type in hypervirulent groups [74]. The outer membrane and the capsule react with the environment and even the human as a host and significantly affect antimicrobial resistance mechanisms (by permeability barrier and efflux pump) and pathogenesis (mainly by the capsule). A relatively unique structure is the rmpA- and/or rmpA2-mediated overproduction of capsular polysaccharides, which contribute to systemic virulence [10,75]. Additionally, the hypervirulent group secretes certain components that significantly affect the environmental survival and the ability of colonization (as a type 6 secretion system) and also the ability to infect humans (as iron-scavenging siderophores) [76].

## 6. Pathogenesis of hvKP

*K. pneumoniae* easily colonizes human skin and mucous membranes, such as the oropharynx and gastrointestinal (GI) tract, where the effects of its propagation are benign and can invade other tissues and cause serious infections in people [77]. In hospital-acquired infections, the invasion of the epithelial or mucosal barriers through surgical wounds, catheters, or endotracheal tubes is the most probable mechanism [68]. However, the infection can be also transmitted through the community and even in healthy individuals with a mechanism other than epithelial or mucosal invasion. The most recorded infection is the liver abscess, which supposes that the hypervirulent spp. can invade the intestinal mucosa to reach the liver through the portal tract [78].

In hvKP strains, genes responsible for colibactin synthesis are found in a mobile element (ICEKp10) that also carries genes for microcin E492 and yersiniabactin production [79,80]. Colibactin is found mainly in the CG23or k1 type of the capsule, but less in the other members of the hypervirulent strain [79,81,82]. In 1928, CG23-I subtypes were found to acquire ICEKp10 and were associated with wide global spread, which suggests that colibactin has a significant effect on the development of new strains [79,80]. Microcin E492 is a bacteriocin with an 8-kDa molecular weight that is potent against the *Enterobacteriaceae* family [83]. Salmochelin attachment is required for activity, as it allows microcin to be taken up by the target bacterium. [84]. So, the production of Salmochelin, microcin E492, and certainly colibactin by a group of hypervirulent strains is considered a significant and effective mechanism for their colonization in the competitive environment of the colon. Glucuronic acid pyruvation and fucose acetylation enable *K. pneumoniae* to overcome phagocytosis [85,86]. Furthermore, after phagocytosis by neutrophils, hvKP with the k1 capsular type can escape from killing mediated by neutrophils; so, the bacteria can transfer and spread to remote sites, like the liver, with the formation of abscess [87]. Bacterial growth both in vivo and in vitro, depending to a great extent on the iron. There are about 12 systems responsible for taking iron in hypervirulent strains. For example, there are three systems based on siderophore (IroA, [88], the Yersinia high-pathogenicity island [88,89], and Iuc [88,90]) and two ABC transporters (Sit and Kfu) [91,92], which form an important part of the hvKP.

## 7. Virulence Factors and Genes

### 7.1. Capsule

**Figure 3:**
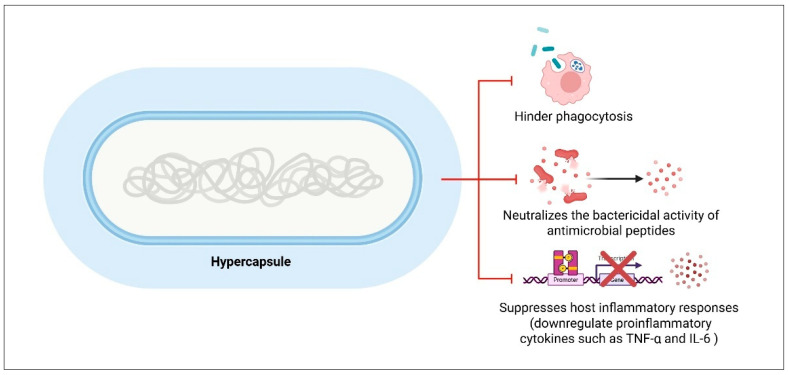
Protective functions of the hypercapsule in hypervirulent *K. pneumoniae* (hvKP). The hypercapsule in hvKP serves as a multifunctional defense structure, contributing to immune evasion and enhanced survival. It impairs phagocytic clearance, reduces susceptibility to host-derived antimicrobial peptides and therapeutic agents, and attenuates inflammatory responses by modulating cytokine production. Created with BioRender.com.

The capsule is a critical virulence factor universally synthesized by *K. pneumoniae* strains. It forms a thick, polysaccharide-rich layer surrounding the bacterial surface, contributing to the organism’s viscous phenotype and playing a vital role in immune evasion. This protective barrier impedes phagocytosis, neutralizes the bactericidal activity of antimicrobial peptides, and suppresses host inflammatory responses by downregulating proinflammatory cytokines such as TNF-α and IL-6 [93,94,95] (Figure 3).

Recent studies have revealed that the hypercapsule of hvKp strains mediates immunosuppression not only by physically shielding bacterial antigens but also by interfering with host pattern recognition receptors (PRRs), such as Toll-like receptors (TLRs) [96]. This interference leads to reduced activation of nuclear factor-kappa B (NF-κB) signaling pathways and diminished transcription of key inflammatory mediators. Additionally, the dense polysaccharide matrix can inhibit complement activation and impair the recruitment of immune effector cells, further attenuating the local inflammatory response [97]. Collectively, these mechanisms contribute to the subdued innate immune response observed during hvKp infections, enabling the bacteria to disseminate systemically with minimal early immune detection.

Hypervirulent *K. pneumoniae* (hvKP) strains typically possess larger and more robust capsules than cKP strains. This hypermucoviscous capsule enhances survival by providing additional protection. However, the enlarged capsule may also act as a physical barrier to horizontal gene transfer by obstructing DNA uptake, potentially explaining the lower prevalence of antimicrobial resistance genes in hvKP compared to cKP strains [98].

Capsule biosynthesis is genetically regulated by chromosomal loci, and due to variations in polysaccharide composition and structural antigens, capsule types are classified into at least 79 serologically distinct K-antigens [99]. Among hvKP strains, eight capsule types are commonly identified: K1, K2, K5, K16, K20, K54, K57, and KN1 [100,101]. K1 and K2 are the most prevalent and are detected in approximately 70% of hvKP isolates [4,21]. Multiple studies have demonstrated that strains with K1 and K2 serotypes exhibit significantly higher virulence compared to those with other capsule types [24,102].

Multilocus sequence typing (MLST) has shown that the K1 serotype is strongly associated with sequence type (ST) 23, whereas the K2 serotype is more genetically diverse, being linked to ST25, ST86, ST375, and ST380 [80,101]. Interestingly, K1 and K2 capsules lack mannose and rhamnose residues—sugar moieties recognized by macrophage lectin receptors—which may reduce phagocytic uptake [103,104].

Additional mechanisms further enhance the virulence of hvKP. In K1 strains, the surface-exposed protein fructose-1,6-bisphosphate aldolase (FBA) has been found to protect neutrophils from cell death under hyperglycemic conditions by upregulating its expression [105]. Moreover, the presence of sialic acid in K1 and K2 capsular polysaccharides contributes to the hypermucoviscous phenotype and is implicated in the antiphagocytic properties of these strains [106,107].

### 7.2. Siderophores

For *K. pneumoniae* to thrive and flourish during infection, it needs iron, a scarce resource that it must obtain from the host. However, as a result of the host’s innate defensive response, a number of iron-binding proteins sequester this metal, preventing a variety of potential pathogens from growing. So, it is not commonly accessible in the host during infection [108,109]. As a result, in order to survive and multiply during infection, *K. pneumoniae* must use tactics to obtain iron from the host. So, *K. pneumoniae* produces siderophores, which are low-molecular-weight iron chelates that consume iron ions from the host’s iron-binding proteins [110].

**Figure 4:**
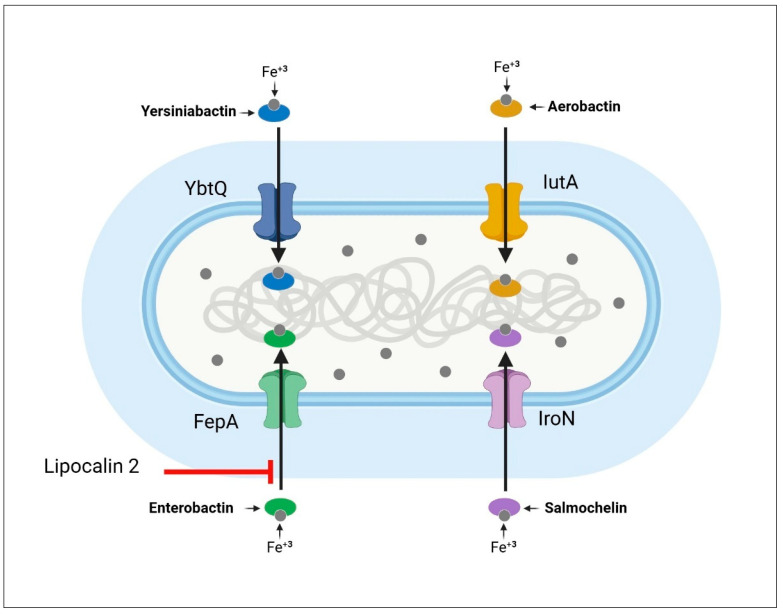
Iron acquisition by siderophores in hypervirulent *Klebsiella pneumoniae* (hvKP). Siderophores enable hvKP to overcome host nutritional immunity by sequestering iron from host iron-binding proteins. With superior iron-binding affinity, these molecules outcompete host proteins and facilitate iron uptake through dedicated bacterial receptors, supporting growth and virulence within the iron-limited environment of the host. Created with BioRender.com.

Siderophores can steal and scavenge iron from host iron-binding proteins because they have a higher affinity for iron than host-binding proteins. The siderophore molecules are subsequently absorbed by the bacterial cell via specific receptors (YbtQ, IutA, FepA, and IroN) [111] (Figure 4). Yersiniabactin (Ybt), enterobactin (Ent), Salmochelin (Iro), and aerobactin (Iuc) are the four types of siderophore molecules produced by hvKP. The last two are apparent in cKP and specific to hvKP. A crucial component of virulence in cKP is yersiniabactin, but no evidence of its importance in hvKP has been discovered [21,88,112]. Aerobactin (Iuc) is the most significant hvKP siderophore system for systemic infection [113] and in laboratory trials, it accounts for nearly 90% of siderophore activity [23]. The host’s natural immunity has the power to reduce their action. For instance, aerobactin can be bound by the antimicrobial peptide LL-37 [114]. Lipocalin-2 protein, another innate immunity component, effectively binds enterobactin, blocking its reuptake by the cell and therefore safeguarding the microorganism [115]. As a result, *K. pneumoniae* produces more than one siderophore to optimize the colonization of different tissues and/or avoid the neutralization of one siderophore by the host [111,116].

### 7.3. Lipopolysaccharide (LPS)

Lipopolysaccharide (LPS), a key structural component of the outer membrane of all gram-negative bacteria, including both cKP and hvKP strains, plays a crucial role in virulence and immune evasion. Structurally, LPS comprises three main regions: lipid A (anchoring the molecule to the bacterial membrane and responsible for endotoxic activity), a core oligosaccharide, and the O-antigen, which extends outward as the most surface-exposed portion. The O-antigen serves as an important shield against the host immune system. It interferes with complement-mediated killing and phagocytosis by binding complement component C3b, thereby preventing membrane attack complex formation and lysis of the bacterial cell [117]. Among the eight known O-antigen serotypes, O1 is the most prevalent in clinical isolates of *K. pneumoniae* and is often associated with increased virulence [74,118].

Experimental evidence suggests that strains lacking the O1 antigen demonstrate reduced pathogenicity compared to O1-positive counterparts [119]. However, in K1 hvKP strains, the contribution of the O1 antigen to virulence remains unclear, likely due to its masking by the overlying hypercapsule. This concealment may limit the O1 antigen’s interaction with host immune components, raising questions about its functional role in the context of hvKP pathogenesis [119,120].

### 7.4. Fimbriae

Fimbriae are filamentous surface structures that facilitate adhesion to host tissues and surfaces, contributing to colonization and biofilm formation in *K. pneumoniae*. Two major types have been experimentally characterized: type 1 fimbriae, which are mannose-sensitive and mediate adhesion to host epithelial cells, and type 3 fimbriae, which are mannose-resistant and primarily associated with biofilm formation on abiotic surfaces. While type 3 fimbriae have been shown to enhance biofilm development in classical strains, their specific role in hvKP remains uncertain [121].

Genomic analyses have identified at least seven distinct fimbrial gene clusters in *K. pneumoniae*—namely, kpa, kpb, kpc, kpd, kpe, kpf, and kfg. Among these, the kpc cluster appears to be the most prevalent in hvKP strains, particularly those of the K1 serotype [122]. Functional studies in strain CG43 revealed that type 3 fimbrial expression and subsequent biofilm formation are regulated by the ferric uptake regulator (Fur) and are influenced by environmental iron levels. This suggests that under iron-limited in vivo conditions—such as those encountered within the host—type 3 fimbriae may play a less significant role in hvKP pathogenicity [4].

### 7.5. Allantoin Metabolism

Most bacterial spp. use allantoin as a nitrogen source, while *K. pneumoniae* uses it as a nitrogen and carbon source [123,124]. Allantoin metabolism in hvKP isolates is controlled by the allantoinase (allB), negative regulator (allR), transcriptional activator (allS), and allantoinpermease (ybbW) enzyme genes [67]. When analyzing operon-holding genes implicated in allantoin metabolism in hvKP strains as compared to cKP, the deletion of allS, an activator of the operon for allantoin metabolism, resulted in a dramatic decrease in the pathogenicity of the hvKP strain in an in vivo test [123]. According to prior research, the allantoin operon is found in larger copy numbers in liver abscess-associated strains than cKP [125].

### 7.6. Colibactin

Colibactin is a genotoxic secondary metabolite produced by *Klebsiella pneumoniae* strains harboring the pks genomic island. This compound is synthesized through the coordinated activity of non-ribosomal peptide synthetases (NRPSs), polyketide synthases (PKSs), and associated tailoring enzymes encoded within the pks cluster [126]. The prevalence of the pks locus is notably high among hypervirulent K1 serotype strains, with detection rates ranging from 66% to 100% in various studies [127].

Although the precise mechanisms by which colibactin contributes to hvKP pathogenesis remain to be fully elucidated, its genotoxic effects are thought to promote host cell damage, thereby facilitating bacterial colonization and dissemination. Colibactin-mediated DNA damage may disrupt host cellular integrity and immune defenses, providing hvKP with a survival advantage during infection [62].

### 7.7. Peg-344

Peg-344 is a genetic marker frequently associated with hvKP and has been identified as a contributor to the enhanced virulence phenotype observed in these strains. Although its exact biochemical function remains unclear, peg-344 is predicted to encode an inner membrane transporter. Experimental evidence from in vivo models suggests that peg-344 is essential for the full expression of hvKP virulence, particularly in pulmonary and hepatic infections. Interestingly, its deletion does not appear to impact the bacteria’s ability to cause systemic infection, indicating that peg-344 may play a more localized role in organ-specific pathogenicity rather than in generalized dissemination [21,128].

### 7.8. Type 6 Secretion System (T6SS)

The type VI secretion system (T6SS) is a specialized molecular apparatus recently identified in hvKP strains. Structurally resembling a contractile nanosyringe, the T6SS is capable of injecting toxic effector proteins directly into competing bacteria or host eukaryotic cells [76]. This contact-dependent mechanism contributes to interbacterial competition and host cell damage, thereby enhancing the bacterium’s ability to colonize and persist within hostile environments. Although still under investigation, the presence of a functional T6SS in hvKP is increasingly recognized as a significant factor in its virulence repertoire.

## 8. Convergence of Hypervirulence and MDR in *K. peumoniae*

There has been an increase in the number of reports that describe the emergence of multidrug-resistant hypervirulent *K. pneumoniae* strains (MDR-hvKP) [129,130]. MDR-hvKP can develop by two mechanisms. Firstly, hvKP strains can acquire antimicrobial resistance genes or plasmids through horizontal transfer and become MDR-hvKP and are known as type I MDR-hvKP. For instance, two carbapenemase plasmids were recovered together from a K2 ST86 MDR-hvKP, bla_NDM-1_-bearing IncN plasmid, and bla_KPC-2_-bearing IncFIIK plasmid [131]. Second, MDR-hvKP strains can be developed by transferring a pLVPK-like virulence plasmid into a classical MDR *K. pneumoniae* strain and are known as type II MDR-hvKP. For instance, consider a lethal outbreak in China triggered by a KPC-producing ST11 strain gaining a pLVPK-like virulence plasmid [68]. In order to quickly diagnose hvKP, including type I and type II MDR-hvKP strains, the *peg-344* gene (transporter in the inner membrane) is present on the virulence plasmid. A *peg-344* loop-mediated isothermal amplification method has also been developed [132].

## 9. The Growing Threat of Antibiotic Resistance in hvKP

*Klebsiella pneumoniae* has developed an alarming capacity for antibiotic resistance, leading the World Health Organization (WHO) to classify it as a “critical” priority pathogen [133] and include it in the WHO priority list [134]. It is a member of the ESKAPE group of pathogens (*Enterococcus faecium*, *Staphylococcus aureus*, *Klebsiella pneumoniae*, *Acinetobacter baumannii*, *Pseudomonas aeruginosa*, and *Enterobacter* spp.), which play a significant role in antibiotic resistance spread in healthcare settings [135].

The bacterium has acquired a wide range of antimicrobial resistance (AMR) genes, primarily through horizontal gene transfer via plasmids, integrons, and transposons. Like other *Enterobacteriaceae*, most of its resistance genes are plasmid-mediated, with *K. pneumoniae* often harboring three or more AMR plasmids that exhibit higher stability than those in *E. coli* [136]. Mobile genetic elements such as integrons (e.g., integron 1), insertion sequences (e.g., IS26), and transposons (e.g., Tn4401a) facilitate the transfer of resistance genes between plasmids and chromosomes [137].

*Klebsiella pneumoniae* exhibits multiple mechanisms of antimicrobial resistance. One primary mechanism is plasmid-mediated resistance, where hvKP strains acquire resistance genes through conjugal plasmids [138,139]. Another major pathway involves the integration of antimicrobial resistance genes, where an integrative and conjugative element (ICE) containing resistance determinants is inserted into a virulence plasmid or chromosome [140,141]. Mutations in chromosomal genes, particularly those encoding outer membrane proteins (OMPs), can also confer resistance by altering drug permeability [142]. Additionally, the convergence of hypervirulence and multidrug resistance occurs when MDR or XDR cKP strains acquire hvKP virulence plasmids, further complicating treatment options [68,142,143].

A proposed mechanism suggests that a conjugal plasmid carrying the KPC carbapenemase gene shares a common 11.2-kb region with some hvKP virulence plasmids, facilitating gene transfer and incorporation. However, further experimental validation is required [142].

All *K. pneumoniae* strains, including hvKP, are intrinsically resistant to ticarcillin and ampicillin, with variable susceptibility to nitrofurantoin. Carbapenemase- or β-lactamase-producing strains carry conjugative plasmids that mediate resistance to tetracyclines, aminoglycosides, fluoroquinolones, and trimethoprim-sulfamethoxazole. Extended-spectrum β-lactamases (ESBLs), such as TEM, CTX-M, and SHV, hydrolyze third- and fourth-generation cephalosporins and aztreonam, leading to decreased susceptibility to β-lactam/β-lactamase inhibitor combinations [29,144]. Some hvKP strains also express ESBL genes [145]. Additionally, certain *K. pneumoniae* plasmids carry *AmpC* β-lactamase genes, which provide resistance similar to ESBLs, but also extend resistance to cephamycins like cefotetan and cefoxitin [144,146].

Carbapenem resistance in *K. pneumoniae* is primarily mediated by carbapenemases, including KPC (Class A), NDM (Class B), IMP (Class B), and OXA (Class D). These enzymes confer resistance to carbapenems, cephamycin, and ESBL-targeted antibiotics. Among them, KPC-type carbapenemases are the most prevalent in *K. pneumoniae* [68,133,147,148,149,150].

Polymyxins, particularly colistin, are considered a last-resort therapy against metallo-β-lactamase-producing *K. pneumoniae* (e.g., NDM-1). However, resistance to polymyxins has emerged through the *mcr-1* gene, which is carried on a stable plasmid [139]. Another resistance mechanism involves the overexpression of the PhoP-PhoQ-Arn pathway, which modifies lipid A to reduce polymyxin binding. The *mgrB* gene regulates this pathway, and its inactivation has been linked to colistin resistance in hvKP [142,150,151].

Resistance to tigecycline has also been documented in *K. pneumoniae* and is associated with the overexpression of *acrR*, a gene regulating efflux pumps, and its regulatory gene *ramA*. Some cKP strains that acquire hvKP virulence plasmids may also develop tigecycline resistance, further complicating treatment strategies [151].

## 10. Clinical and Epidemiological Risk Factors

The presence of K1 or K2 serotypes and virulence factors does not appear to influence mortality. However, several clinical factors, including bacteremia, respiratory distress (respiratory rate > 30 breaths/min), altered mental status, septic shock, arterial pH < 7.35, tachycardia (heart rate > 125 beats/min), hematocrit < 30%, and elevated blood urea nitrogen (>10.71 mmol/L), have been identified as significant risk factors for increased mortality [152].

The mortality risk is comparable between infections caused by hvKP and cKP. However, cKP infections are more prevalent among hospitalized cancer patients, likely due to their immunocompromised state [153].

Patients with hypervirulent bloodstream infections (hvKP-BSIs) are more likely to have diabetes mellitus (DM) but less likely to be immunosuppressed compared to those with cKP-BSIs. Pyogenic liver abscesses are a more common cause of hvKP-BSIs than cKP-BSIs. Studies have identified DM, community-acquired infections, solid malignancies, and hypertension as substantial risk factors for hvKP infections, while immunosuppression appears to be a protective factor against hvKP-BSIs [66,154,155].

Although hvKP infections can affect all ethnic groups, they are more frequently reported in populations from Asia, the Hispanic community, and Pacific Island nations, likely due to increased exposure and colonization rates [5,7,57,156,157,158,159,160,161].

Regarding gender-based susceptibility, some studies suggest that males are more likely to acquire hvKP infections than females [158]. However, other studies have reported no significant difference in infection rates between genders [52].

## 11. Association with Malignancy

Many studies have reported the increased incidence of cancer colon in patients with a liver abscess caused by *K. pneumoniae* in comparison to those without a liver abscess or even with a liver abscess caused by pathogens other than *K. pneumoniae* [162,163]. Colibactin is a genotoxin produced by some spp. of *Klebsiella* and it is thought to be the causative factor. Other studies suppose that a liver abscess due to the virulent spp. of klebsiella is the precancerous lesion [164,165]. Colonoscopy should be taken into consideration as a screening tool for all individuals with a liver abscess caused by the hypervirulent spp. of klebsiella, especially if they have a risk factor (e.g., family history or age).

## 12. Clinical Challenges with HVKP Infection

### 12.1. Diagnostic Challenges

One of the primary challenges in managing hvKP infections is accurate strain identification. In China, hvKP was detected in 22.8% (84/369) of *K. pneumoniae* clinical isolates associated with invasive infections [138,166]. Compared to cKP, hvKP strains are more likely to cause widespread invasive infections, leading to increased mortality. However, there is no universally accepted definition of hvKP in clinical practice, and different studies have employed varying criteria for classification.

Traditional diagnostic methods, such as the string test and aerobactin test, have proven unreliable, making it difficult for clinicians to identify hvKP strains and interpret test results accurately [31,66,138,167]. Diagnosis is often based on clinical suspicion, as hvKP is frequently associated with metastatic invasive infections in otherwise healthy individuals. Initially believed to predominantly affect young adults, hvKP infections have also been found to be prevalent among older patients, with a study by Liu et al. reporting a significant occurrence rate of 45.7%, further complicating clinical identification [168].

Genotypic and phenotypic markers provide a more accurate means of differentiating hvKP from cKP. Hypervirulence-associated genetic markers, such as those found on mobile genetic elements and virulence plasmids, have been extensively studied [138]. Key biomarkers, including sequence type (ST), capsular serotype, and virulence-associated genes (*iroB*, *peg-344*, *iucA*, *prmpA2*, and *rmpA*), have shown promise in distinguishing hvKP strains from cKP [167]. According to Russo et al., *iuc* and total siderophore production, along with *rmpA* and *rmpA2*, may serve as the most sensitive and reliable markers for hvKP identification [21]. However, the widespread implementation of these molecular diagnostic tools remains limited due to economic and logistical constraints, making clinical differentiation between hvKP and cKP a persistent challenge.

### 12.2. Antibiotic Resistance and Treatment Challenges

A significant hurdle in hvKP management is its growing resistance to antibiotics. Most hvKP strains exhibit resistance to multiple antimicrobial agents, further complicating treatment [138]. The frequency of antibiotic-resistant hvKP isolates has increased in recent years, particularly in regions where hvKP is endemic, such as China [31,68,138,144]. A study by Zang et al. reported that 37% of clinical *K. pneumoniae* isolates were classified as hvKP due to the presence of *rmpA*, and 13% of these strains had acquired ESBL production capabilities [31].

The acquisition of antibiotic resistance in hvKP occurs through multiple mechanisms, including horizontal gene transfer and the acquisition of plasmids encoding resistance determinants [11]. The convergence of hypervirulence and MDR significantly complicates treatment, as conventional antibiotic regimens often prove ineffective. The limited therapeutic options highlight the urgent need for alternative treatment strategies, such as phage therapy, combination antibiotic therapy, and novel antimicrobial agents [169,170].

Given these challenges, strict infection control measures and surveillance programs are essential to prevent the spread of hvKP in healthcare settings. Comprehensive antimicrobial stewardship programs should be reinforced to curb antibiotic overuse, which drives resistance development. Addressing hvKP infections requires an integrated approach that combines rapid diagnostics, effective antimicrobial strategies, and stringent infection prevention protocols.

## 13. Controlling the hvKP Infection: Recommendations

A multifaceted approach is essential for controlling hvKP infections, incorporating both traditional and innovative strategies. Key measures include infection control and monitoring, antibiotic therapy, phage therapy, biofilm-disrupting agents, monoclonal antibodies, and vaccine development [113,171,172].

Clinical Management: Radiological methods and abscess drainage are critical, particularly given the frequent development of abscesses in hvKP infections. The hypermucoviscous (HMV) phenotype often leads to highly viscous abscess fluid, complicating management. Antimicrobial therapy is the primary treatment for abscesses smaller than 5 cm, while larger abscesses typically require drainage [113,173]. Given reports of reinfection occurring months or even years post-treatment, sustained long-term follow-up is essential to ensure effective disease management [4].

Empirical antibiotic therapy should be guided by local resistance patterns. While *K. pneumoniae* is inherently resistant to ampicillin [154], no definitive treatment regimen exists for hypervirulent subtypes. However, meropenem in combination with ceftriaxone is suggested for CNS infections, while ocular infections may require a combination of systemic and local treatment with cefazolin, ceftazidime, aminoglycosides, and imipenem [113]. Treatment duration typically spans six weeks, depending on the extent and site of infection [4,158].

Emerging Therapeutic Strategies: Phage therapy has demonstrated potential in combination with antibiotics for treating hvKP infections and preventing the spread of carbapenem-resistant strains [133,174,175]. Recent research has identified phages with activity against multidrug-resistant *K. pneumoniae* [176], highlighting their potential as adjunctive therapy.

Bioconjugate vaccines offer another promising avenue for infection prevention. A bivalent bioconjugate vaccine targeting K1/K2 *K. pneumoniae* has been successfully developed using genetically engineered *E. coli* strains [177]. Additionally, vaccines incorporating B cell epitopes from type 1 fimbriae antigens have shown potential for protective immunity [178].

Monoclonal antibody therapies have also been explored as a means to reduce hvKP transmission. In experimental models, monoclonal antibodies significantly decreased the bacterial load in the intestine, preventing systemic dissemination [172,179].

Biofilm-Targeted Approaches: Biofilm formation is a major contributor to hvKP persistence and antibiotic resistance, providing a protective barrier that enhances bacterial survival and limits antibiotic penetration [180]. Effective strategies for biofilm disruption include quorum-sensing (QS) inhibitors, such as furans, pyridines, phenyl acyl alkaloids, and fatty acids, which interfere with bacterial communication and biofilm maturation. Enzymatic approaches, such as dispersin B and DNase, degrade the extracellular polymeric substances that maintain biofilm structure, enhancing bacterial susceptibility to antibiotics [181].

Additionally, synthetic polymers and antimicrobial peptides, including polymyxin and polyalanine, have shown promise in preventing biofilm formation and promoting biofilm degradation [182]. Metal-based therapies utilizing copper and gold nanoparticles exhibit antimicrobial properties by disrupting bacterial membranes and interfering with metabolic processes [183,184]. Moreover, combining multiple biofilm-disrupting agents has demonstrated synergistic effects, offering a promising avenue for improved hvKP eradication [157].

Future research must prioritize the development of rapid diagnostic tools to efficiently identify hvKP strains and their resistance profiles. Whole-genome sequencing and machine learning-based predictive models should be explored to enhance early detection and epidemiological tracking.

Preventative measures, including vaccine development and monoclonal antibody therapies, require further refinement to provide long-term protection against hvKP infections. Efforts should also focus on enhancing biofilm-disrupting agents to improve treatment outcomes in persistent infections.

Ultimately, a global collaborative approach integrating antimicrobial stewardship, infection control, and interdisciplinary research will be essential in addressing the rising threat of hvKP infections.

## 14. Conclusions

*Klebsiella pneumoniae* remains one of the most prevalent causes of nosocomial infections, particularly in immunocompromised patients. The emergence of hypervirulent subtypes poses a significant global health threat, as these strains have expanded the range of susceptible individuals to include both immunocompromised and otherwise healthy individuals. hvKP is capable of causing severe and often fatal infections across multiple organ systems, with hepatic abscesses being among the first reported manifestations.

Distinguishing between cKP and hvKP is critical, particularly through the identification of specific biomarkers that facilitate accurate diagnosis. Moreover, hvKP can acquire a broad range of antibiotic-resistance genes due to its widespread presence in both the human body and the environment. The WHO has classified *K. pneumoniae* as a critical pathogen due to its increasing resistance to antibiotics. The widespread dissemination of resistance genes has led to the rapid emergence of MDR and XDR hvKP strains, which are resistant to most conventional antibiotics.

To combat hvKP infections, several novel strategies have been explored, including phage therapy, biofilm-disrupting agents, monoclonal antibodies, and vaccine development. Future research should focus on advancing these therapeutic approaches while also considering the potential applications of nanotechnology in controlling hvKP infections. A concerted global effort is necessary to develop effective interventions and mitigate the growing threat posed by this highly virulent and antibiotic-resistant pathogen.

## Data Availability

Not applicable.

## References

[1] Magill S.S., Edwards J.R., Bamberg W., Beldavs Z.G., Dumyati G., Kainer M.A., Lynfield R., Maloney M., McAllister-Hollod L., Nadle J. (2014). Multistate point-prevalence survey of health care–associated infections. N. Engl. J. Med..

[2] Paczosa M.K., Mecsas J. (2016). *Klebsiella pneumoniae*: Going on the Offense with a Strong Defense. Microbiol. Mol. Biol. Rev. MMBR.

[3] Makharita R.R., El-Kholy I., Hetta H.F., Abdelaziz M.H., Hagagy F.I., Ahmed A.A., Algammal A.M. (2020). Antibiogram and genetic characterization of carbapenem-resistant gram-negative pathogens incriminated in healthcare-associated infections. Infect. Drug Resist..

[4] Shon A.S., Bajwa R.P., Russo T.A. (2013). Hypervirulent (hypermucoviscous) *Klebsiella pneumoniae*: A new and dangerous breed. Virulence.

[5] Fazili T., Sharngoe C., Endy T., Kiska D., Javaid W., Polhemus M. (2016). *Klebsiella pneumoniae* liver abscess: An emerging disease. Am. J. Med. Sci..

[6] Lin Y.-T., Siu L.K., Lin J.-C., Chen T.-L., Tseng C.-P., Yeh K.-M., Chang F.-Y., Fung C.-P. (2012). Seroepidemiology of *Klebsiella pneumoniae* colonizing the intestinal tract of healthy Chinese and overseas Chinese adults in Asian countries. BMC Microbiol..

[7] Nadasy K.A., Domiati-Saad R., Tribble M.A. (2007). Invasive *Klebsiella pneumoniae* syndrome in North America. Clin. Infect. Dis..

[8] Al Ismail D., Campos-Madueno E.I., Donà V., Endimiani A. (2024). Hypervirulent *Klebsiella pneumoniae* (hvKp): Overview, Epidemiology, and Laboratory Detection. Pathog. Immun..

[9] Kareem S.M., Al-Kadmy I.M., Kazaal S.S., Mohammed Ali A.N., Aziz S.N., Makharita R.R., Algammal A.M., Al-Rejaie S., Behl T., Batiha G.E.-S. (2021). Detection of gyrA and parC mutations and prevalence of plasmid-mediated quinolone resistance genes in *Klebsiella pneumoniae*. Infect. Drug Resist..

[10] Fang C.-T., Chuang Y.-P., Shun C.-T., Chang S.-C., Wang J.-T. (2004). A novel virulence gene in *Klebsiella pneumoniae* strains causing primary liver abscess and septic metastatic complications. J. Exp. Med..

[11] Russo T.A., Marr C.M. (2019). Hypervirulent *Klebsiella pneumoniae*. Clin. Microbiol. Rev..

[12] Mai D., Wu A., Li R., Cai D., Tong H., Wang N., Tan J. (2023). Identification of hypervirulent *Klebsiella pneumoniae* based on biomarkers and Galleria mellonella infection model. BMC Microbiol..

[13] Nannini E.C., Lahitte M., Scapellato P., Nemirosvky C., Zylberman M., Vila A., Rodríguez V., Zucchi R., Mykietiuk A., David V. (2025). Diversity of hypervirulent *Klebsiella pneumoniae* clones causing cryptogenic liver abscesses and metastatic complications in Argentina. Rev. Argent. Microbiol..

[14] Pu D., Zhao J., Chang K., Zhuo X., Cao B. (2023). “Superbugs” with hypervirulence and carbapenem resistance in *Klebsiella pneumoniae*: The rise of such emerging nosocomial pathogens in China. Sci. Bull..

[15] Chen T., Ying L., Xiong L., Wang X., Lu P., Wang Y., Shen P., Xiao Y. (2024). Understanding carbapenem-resistant hypervirulent *Klebsiella pneumoniae*: Key virulence factors and evolutionary convergence. hLife.

[16] García-Cobos S., Oteo-Iglesias J., Pérez-Vázquez M. (2025). Hypervirulent *Klebsiella pneumoniae*: Epidemiology outside Asian countries, antibiotic resistance association, methods of detection and clinical management. Enfermedades Infecc. Microbiol. Clín..

[17] Algammal A., Hetta H.F., Mabrok M., Behzadi P. (2023). Editorial: Emerging multidrug-resistant bacterial pathogens “superbugs”: A rising public health threat. Front. Microbiol..

[18] Chew K.L., Lin R.T., Teo J.W. (2017). *Klebsiella pneumoniae* in Singapore: Hypervirulent infections and the carbapenemase threat. Front. Cell. Infect. Microbiol..

[19] Catalán-Nájera J.C., Garza-Ramos U., Barrios-Camacho H. (2017). Hypervirulence and hypermucoviscosity: Two different but complementary *Klebsiella* spp. phenotypes?. Virulence.

[20] Zhu J., Wang T., Chen L., Du H. (2021). Virulence factors in hypervirulent *Klebsiella pneumoniae*. Front. Microbiol..

[21] Russo T.A., Olson R., Fang C.-T., Stoesser N., Miller M., MacDonald U., Hutson A., Barker J.H., La Hoz R.M., Johnson J.R. (2018). Identification of biomarkers for differentiation of hypervirulent *Klebsiella pneumoniae* from classical *K. pneumoniae*. J. Clin. Microbiol..

[22] Yu W.-L., Ko W.-C., Cheng K.-C., Lee C.-C., Lai C.-C., Chuang Y.-C. (2008). Comparison of prevalence of virulence factors for *Klebsiella pneumoniae* liver abscesses between isolates with capsular K1/K2 and non-K1/K2 serotypes. Diagn. Microbiol. Infect. Dis..

[23] Russo T.A., Olson R., MacDonald U., Beanan J., Davidson B.A. (2015). Aerobactin, but not yersiniabactin, salmochelin, or enterobactin, enables the growth/survival of hypervirulent (hypermucoviscous) *Klebsiella pneumoniae* ex vivo and in vivo. Infect. Immun..

[24] Wang L., Shen D., Wu H., Ma Y. (2017). Resistance of hypervirulent *Klebsiella pneumoniae* to both intracellular and extracellular killing of neutrophils. PLoS ONE.

[25] Walker K.A., Treat L.P., Sepúlveda V.E., Miller V.L. (2020). The small protein RmpD drives hypermucoviscosity in *Klebsiella pneumoniae*. MBio.

[26] Liu Y.-C., Cheng D.-L., Lin C.-L. (1986). *Klebsiella pneumoniae* liver abscess associated with septic endophthalmitis. Arch. Intern. Med..

[27] Liu Y.-C., Yen M.-Y., Wang R.-S. (1991). Septic metastatic lesions of pyogenic liver abscess: Their association with *Klebsiella pneumoniae* bacteremia in diabetic patients. Arch. Intern. Med..

[28] Wang J.-H., Liu Y.-C., Lee S.S.-J., Yen M.-Y., Chen Y.-S., Wang J.-H., Wann S.-R., Lin H.-H. (1998). Primary liver abscess due to *Klebsiella pneumoniae* in Taiwan. Clin. Infect. Dis..

[29] Kong H., Yu F., Zhang W., Li X. (2017). Clinical and microbiological characteristics of pyogenic liver abscess in a tertiary hospital in East China. Medicine.

[30] Oikonomou K.G., Aye M. (2017). *Klebsiella pneumoniae* liver abscess: A case series of six Asian patients. Am. J. Case Rep..

[31] Zhang Y., Zhao C., Wang Q., Wang X., Chen H., Li H., Zhang F., Li S., Wang R., Wang H. (2016). High Prevalence of Hypervirulent *Klebsiella pneumoniae* Infection in China: Geographic Distribution, Clinical Characteristics, and Antimicrobial Resistance. Antimicrob. Agents Chemother..

[32] Marrie T.J., Durant H., Yates L. (1989). Community-acquired pneumonia requiring hospitalization: 5-year prospective study. Rev. Infect. Dis..

[33] Carpenter J.L. (1990). Klebsiella pulmonary infections: Occurrence at one medical center and review. Rev. Infect. Dis..

[34] Vergis E.N., Indorf A., File T.M., Phillips J., Bates J., Tan J., Sarosi G.A., Grayston J.T., Summersgill J., Victor L.Y. (2000). Azithromycin vs cefuroxime plus erythromycin for empirical treatment of community-acquired pneumonia in hospitalized patients: A prospective, randomized, multicenter trial. Arch. Intern. Med..

[35] Ko W.-C., Paterson D.L., Sagnimeni A.J., Hansen D.S., Von Gottberg A., Mohapatra S., Casellas J.M., Goossens H., Mulazimoglu L., Trenholme G. (2002). Community-acquired *Klebsiella pneumoniae* bacteremia: Global differences in clinical patterns. Emerg. Infect. Dis..

[36] Lee H.C., Chuang Y.C., Yu W.L., Lee N.Y., Chang C.M., Ko N.Y., Wang L.R., Ko W.C. (2006). Clinical implications of hypermucoviscosity phenotype in *Klebsiella pneumoniae* isolates: Association with invasive syndrome in patients with community-acquired bacteraemia. J. Intern. Med..

[37] Kishibe S., Okubo Y., Morino S., Hirotaki S., Tame T., Aoki K., Ishii Y., Ota N., Shimomura S., Sakakibara H. (2016). Pediatric hypervirulent *Klebsiella pneumoniae* septic arthritis. Pediatr. Int..

[38] Chang W.-N., Huang C.-R., Lu C.-H., Chien C.-C. (2012). Adult *Klebsiella pneumoniae* meningitis in Taiwan: An overview. Acta Neurol Taiwan.

[39] Tang L.-M., Chen S.-T., Hsu W.-C., Chen C.-M. (1997). Klebsiella meningitis in Taiwan: An overview. Epidemiol. Infect..

[40] Jang T., Wang F., Wang L., Yu K., Liu C. (1993). Gram-negative bacillary meningitis in adults: A recent six-year experience. J. Formos. Med. Assoc. = Taiwan Yi Zhi.

[41] Fang C., Chang S.-C., Hsueh P., Chen Y., Sau W., Luh K. (2000). Microbiologic features of adult community-acquired bacterial meningitis in Taiwan. J. Formos. Med. Assoc. = Taiwan Yi Zhi.

[42] Durand M.L., Calderwood S.B., Weber D.J., Miller S.I., Southwick F.S., Caviness Jr V.S., Swartz M.N. (1993). Acute bacterial meningitis in adults--A review of 493 episodes. N. Engl. J. Med..

[43] Lin Y.-T., Liu C.-J., Chen T.-J., Fung C.-P. (2012). Long-term mortality of patients with septic ocular or central nervous system complications from pyogenic liver abscess: A population-based study. PLoS ONE.

[44] Fang C.-T., Chen Y.-C., Chang S.-C., Sau W.-Y., Luh K.-T. (2000). *Klebsiella pneumoniae* meningitis: Timing of antimicrobial therapy and prognosis. Qjm.

[45] Yoon W. (2013). A rare presentation of brain abscess and ventriculitis/INS; caused by Klebsiella pneumonia. J. Neurol. Sci..

[46] Hentzien M., Rosman J., Decré D., Brenkle K., Mendes-Martins L., Mateu P. (2017). Seven hypervirulent ST380 *Klebsiella pneumoniae* septic localizations. Med. Mal. Infect..

[47] Hsieh M.-J., Lu T.-C., Ma M.H.-M., Wang H.-P., Chen S.-C. (2009). Unrecognized cervical spinal epidural abscess associated with metastatic *Klebsiella pneumoniae* bacteremia and liver abscess in nondiabetic patients. Diagn. Microbiol. Infect. Dis..

[48] Doud M.S., Grimes-Zeppegno R., Molina E., Miller N., Balachandar D., Schneper L., Poppiti R., Mathee K. (2009). A k2A-positive *Klebsiella pneumoniae* causes liver and brain abscess in a Saint Kitt's man. Int. J. Med. Sci..

[49] Kuramochi G., Takei S.-i., Sato M., Isokawa O., Takemae T., Takahashi A. (2005). *Klebsiella pneumoniae* liver abscess associated with septic spinal epidural abscess. Hepatol. Res..

[50] Cheng N.-C., Yu Y.-C., Tai H.-C., Hsueh P.-R., Chang S.-C., Lai S.-Y., Yi W.-C., Fang C.-T. (2012). Recent trend of necrotizing fasciitis in Taiwan: Focus on monomicrobial *Klebsiella pneumoniae* necrotizing fasciitis. Clin. Infect. Dis..

[51] Gunnarsson G.L., Brandt P.B., Gad D., Struve C., Justesen U.S. (2009). Monomicrobial necrotizing fasciitis in a white male caused by hypermucoviscous *Klebsiella pneumoniae*. J. Med. Microbiol..

[52] Ye M., Tu J., Jiang J., Bi Y., You W., Zhang Y., Ren J., Zhu T., Cao Z., Yu Z. (2016). Clinical and genomic analysis of liver abscess-causing *Klebsiella pneumoniae* identifies new liver abscess-associated virulence genes. Front. Cell. Infect. Microbiol..

[53] Ng D., Frazee B. (2015). Necrotizing fasciitis caused by hypermucoviscous *Klebsiella pneumoniae* in a Filipino female in North America. West. J. Emerg. Med..

[54] Mgbemena O., Serota D.P., Kumar S., Wozniak J.E., Weiss D.S., Kempker R.R. (2017). Peculiar purulence: Hypervirulent *Klebsiella pneumoniae* causing pyomyositis. Int. J. Infect. Dis..

[55] Prokesch B.C., TeKippe M., Kim J., Raj P., TeKippe E.M., Greenberg D.E. (2016). Primary osteomyelitis caused by hypervirulent *Klebsiella pneumoniae*. Lancet Infect. Dis..

[56] Frazee B.W., Hansen S., Lambert L. (2009). Invasive infection with hypermucoviscous *Klebsiella pneumoniae*: Multiple cases presenting to a single emergency department in the United States. Ann. Emerg. Med..

[57] McCabe R., Lambert L., Frazee B. (2010). Invasive *Klebsiella pneumoniae* infections, California, USA. Emerg. Infect. Dis..

[58] Paraschiv F., Popescu G.A., Borcan A.M. (2018). Septic cutaneous emboli revealing a severe case of *Klebsiella pneumoniae* liver abscess syndrome. JMM Case Rep..

[59] Qu K., Liu C., Wang Z.-X., Tian F., Wei J.-C., Tai M.-H., Zhou L., Meng F.-D., Wang R.-T., Xu X.-S. (2012). Pyogenic liver abscesses associated with nonmetastatic colorectal cancers: An increasing problem in Eastern Asia. World J. Gastroenterol. WJG.

[60] Rivero A., Gomez E., Alland D., Huang D.B., Chiang T. (2010). K2 serotype *Klebsiella pneumoniae* causing a liver abscess associated with infective endocarditis. J. Clin. Microbiol..

[61] Balestrino D., Ghigo J.M., Charbonnel N., Haagensen J.A., Forestier C. (2008). The characterization of functions involved in the establishment and maturation of *Klebsiella pneumoniae* in vitro biofilm reveals dual roles for surface exopolysaccharides. Environ. Microbiol..

[62] Lam M., Wyres K.L., Judd L.M., Wick R.R., Jenney A., Brisse S., Holt K.E. (2018). Tracking key virulence loci encoding aerobactin and salmochelin siderophore synthesis in *Klebsiella pneumoniae*. Genome Med..

[63] Turton J.F., Englender H., Gabriel S.N., Turton S.E., Kaufmann M.E., Pitt T.L. (2007). Genetically similar isolates of *Klebsiella pneumoniae* serotype K1 causing liver abscesses in three continents. J. Med. Microbiol..

[64] Karlsson M., Stanton R.A., Ansari U., McAllister G., Chan M.Y., Sula E., Grass J.E., Duffy N., Anacker M.L., Witwer M.L. (2019). Identification of a carbapenemase-producing hypervirulent *Klebsiella pneumoniae* isolate in the United States. Antimicrob. Agents Chemother..

[65] Kain M.J.W., Reece N.L., Parry C.M., Rajahram G.S., Paterson D.L., Woolley S.D. (2024). The rapid emergence of hypervirulent klebsiella species and burkholderia pseudomallei as major health threats in southeast Asia: The urgent need for recognition as neglected tropical diseases. Trop. Med. Infect. Dis..

[66] Guo Y., Wang S., Zhan L., Jin Y., Duan J., Hao Z., Lv J., Qi X., Chen L., Kreiswirth B.N. (2017). Microbiological and clinical characteristics of hypermucoviscous *Klebsiella pneumoniae* isolates associated with invasive infections in China. Front. Cell. Infect. Microbiol..

[67] Shankar C., Veeraraghavan B., Nabarro L.E.B., Ravi R., Ragupathi N.K.D., Rupali P. (2018). Whole genome analysis of hypervirulent *Klebsiella pneumoniae* isolates from community and hospital acquired bloodstream infection. BMC Microbiol..

[68] Gu D., Dong N., Zheng Z., Lin D., Huang M., Wang L., Chan E.W.-C., Shu L., Yu J., Zhang R. (2018). A fatal outbreak of ST11 carbapenem-resistant hypervirulent *Klebsiella pneumoniae* in a Chinese hospital: A molecular epidemiological study. Lancet Infect. Dis..

[69] Tang M., Kong X., Hao J., Liu J. (2020). Epidemiological characteristics and formation mechanisms of multidrug-resistant hypervirulent *Klebsiella pneumoniae*. Front. Microbiol..

[70] WHO Antimicrobial Resistance, Hypervirulent Klebsiella pneumoniae—Global Situation. https://www.who.int/emergencies/disease-outbreak-news/item/2024-DON527.

[71] Fang C.-T., Lai S.-Y., Yi W.-C., Hsueh P.-R., Liu K.-L., Chang S.-C. (2007). *Klebsiella pneumoniae* genotype K1: An emerging pathogen that causes septic ocular or central nervous system complications from pyogenic liver abscess. Clin. Infect. Dis..

[72] Liu Y., Liu P.-p., Wang L.-h., Wei D.-d., Wan L.-G., Zhang W. (2017). Capsular polysaccharide types and virulence-related traits of epidemic KPC-producing *Klebsiella pneumoniae* isolates in a Chinese University Hospital. Microb. Drug Resist..

[73] Yu F., Lv J., Niu S., Du H., Tang Y.-W., Bonomo R.A., Kreiswirth B.N., Chen L. (2018). In vitro activity of ceftazidime-avibactam against carbapenem-resistant and hypervirulent *Klebsiella pneumoniae* isolates. Antimicrob. Agents Chemother..

[74] Follador R., Heinz E., Wyres K.L., Ellington M.J., Kowarik M., Holt K.E., Thomson N.R. (2016). The diversity of *Klebsiella pneumoniae* surface polysaccharides. Microb. Genom..

[75] Cheng H., Chen Y., Wu C., Chang H., Lai Y., Peng H.-L. (2010). RmpA regulation of capsular polysaccharide biosynthesis in *Klebsiella pneumoniae* CG43. J. Bacteriol..

[76] Sarris P.F., Zoumadakis C., Panopoulos N.J., Scoulica E.V. (2011). Distribution of the putative type VI secretion system core genes in Klebsiella spp. Infect. Genet. Evol..

[77] Dao T.T., Liebenthal D., Tran T.K., Ngoc Thi Vu B., Ngoc Thi Nguyen D., Thi Tran H.K., Thi Nguyen C.K., Thi Vu H.L., Fox A., Horby P. (2014). *Klebsiella pneumoniae* oropharyngeal carriage in rural and urban Vietnam and the effect of alcohol consumption. PLoS ONE.

[78] Spadoni I., Zagato E., Bertocchi A., Paolinelli R., Hot E., Di Sabatino A., Caprioli F., Bottiglieri L., Oldani A., Viale G. (2015). A gut-vascular barrier controls the systemic dissemination of bacteria. Science.

[79] Lam M., Wyres K.L., Duchêne S., Wick R.R., Judd L.M., Gan Y.-H., Hoh C.-H., Archuleta S., Molton J.S., Kalimuddin S. (2018). Population genomics of hypervirulent *Klebsiella pneumoniae* clonal-group 23 reveals early emergence and rapid global dissemination. Nat. Commun..

[80] Struve C., Roe C.C., Stegger M., Stahlhut S.G., Hansen D.S., Engelthaler D.M., Andersen P.S., Driebe E.M., Keim P., Krogfelt K.A. (2015). Mapping the evolution of hypervirulent *Klebsiella pneumoniae*. MBio.

[81] Lai Y.-C., Lin A.-C., Chiang M.-K., Dai Y.-H., Hsu C.-C., Lu M.-C., Liau C.-Y., Chen Y.-T. (2014). Genotoxic *Klebsiella pneumoniae* in Taiwan. PLoS ONE.

[82] Chen Y.-T., Lai Y.-C., Tan M.-C., Hsieh L.-Y., Wang J.-T., Shiau Y.-R., Wang H.-Y., Lin A.-C., Lai J.-F., Huang I.-W. (2017). Prevalence and characteristics of pks genotoxin gene cluster-positive clinical *Klebsiella pneumoniae* isolates in Taiwan. Sci. Rep..

[83] de Lorenzo V. (1984). Isolation and characterization of microcin E 492 from *Klebsiella pneumoniae*. Arch. Microbiol..

[84] Lagos R., Baeza M., Corsini G., Hetz C., Strahsburger E., Castillo J.A., Vergara C., Monasterio O. (2001). Structure, organization and characterization of the gene cluster involved in the production of microcin E492, a channel-forming bacteriocin. Mol. Microbiol..

[85] Sá-Pessoa J., Przybyszewska K., Vasconcelos F.N., Dumigan A., Frank C.G., Hobley L., Bengoechea J.A. (2020). *Klebsiella pneumoniae* reduces SUMOylation to limit host defense responses. Mbio.

[86] Pan P.-C., Chen H.-W., Wu P.-K., Wu Y.-Y., Lin C.-H., Wu J.H. (2011). Mutation in fucose synthesis gene of *Klebsiella pneumoniae* affects capsule composition and virulence in mice. Exp. Biol. Med..

[87] Wu J.H., Wu A.M., Tsai C.G., Chang X.-Y., Tsai S.-F., Wu T.-S. (2008). Contribution of fucose-containing capsules in *Klebsiella pneumoniae* to bacterial virulence in mice. Exp. Biol. Med..

[88] Hsieh P.-F., Lin T.-L., Lee C.-Z., Tsai S.-F., Wang J.-T. (2008). Serum-induced iron-acquisition systems and TonB contribute to virulence in *Klebsiella pneumoniae* causing primary pyogenic liver abscess. J. Infect. Dis..

[89] Lawlor M.S., O'connor C., Miller V.L. (2007). Yersiniabactin is a virulence factor for *Klebsiella pneumoniae* during pulmonary infection. Infect. Immun..

[90] Nassif X., Sansonetti P.J. (1986). Correlation of the virulence of *Klebsiella pneumoniae* K1 and K2 with the presence of a plasmid encoding aerobactin. Infect. Immun..

[91] Ma L.-C., Fang C.-T., Lee C.-Z., Shun C.-T., Wang J.-T. (2005). Genomic heterogeneity in *Klebsiella pneumoniae* strains is associated with primary pyogenic liver abscess and metastatic infection. J. Infect. Dis..

[92] Sun W.-S.W., Syu W.-J., Ho W.-L., Lin C.-N., Tsai S.-F., Wang S.-H. (2014). SitA contributes to the virulence of *Klebsiella pneumoniae* in a mouse infection model. Microbes Infect..

[93] Cortés G., Borrell N., de Astorza B., Gómez C., Sauleda J., Albertí S. (2002). Molecular analysis of the contribution of the capsular polysaccharide and the lipopolysaccharide O side chain to the virulence of *Klebsiella pneumoniae* in a murine model of pneumonia. Infect. Immun..

[94] Domenico P., Salo R.J., Cross A.S., Cunha B.A. (1994). Polysaccharide capsule-mediated resistance to opsonophagocytosis in *Klebsiella pneumoniae*. Infect. Immun..

[95] Yoshida K., Matsumoto T., Tateda K., Uchida K., Tsujimoto S., Yamaguchi K. (2000). Role of bacterial capsule in local and systemic inflammatory responses of mice during pulmonary infection with *Klebsiella pneumoniae*. J. Med. Microbiol..

[96] Xu Q., Yang X., Chan E.W.C., Chen S. (2021). The hypermucoviscosity of hypervirulent *K. pneumoniae* confers the ability to evade neutrophil-mediated phagocytosis. Virulence.

[97] Liu X., Xu Q., Yang X., Heng H., Yang C., Yang G., Peng M., Chan E.-C., Chen S. (2025). Capsular polysaccharide enables *Klebsiella pneumoniae* to evade phagocytosis by blocking host-bacteria interactions. mBio.

[98] Wyres K.L., Wick R.R., Judd L.M., Froumine R., Tokolyi A., Gorrie C.L., Lam M.M., Duchêne S., Jenney A., Holt K.E. (2019). Distinct evolutionary dynamics of horizontal gene transfer in drug resistant and virulent clones of *Klebsiella pneumoniae*. PLoS Genet..

[99] Pan Y.-J., Lin T.-L., Chen C.-T., Chen Y.-Y., Hsieh P.-F., Hsu C.-R., Wu M.-C., Wang J.-T. (2015). Genetic analysis of capsular polysaccharide synthesis gene clusters in 79 capsular types of *Klebsiella* spp. Sci. Rep..

[100] Pan Y.-J., Fang H.-C., Yang H.-C., Lin T.-L., Hsieh P.-F., Tsai F.-C., Keynan Y., Wang J.-T. (2008). Capsular polysaccharide synthesis regions in *Klebsiella pneumoniae* serotype K57 and a new capsular serotype. J. Clin. Microbiol..

[101] Lee I.R., Molton J.S., Wyres K.L., Gorrie C., Wong J., Hoh C.H., Teo J., Kalimuddin S., Lye D.C., Archuleta S. (2016). Differential host susceptibility and bacterial virulence factors driving Klebsiella liver abscess in an ethnically diverse population. Sci. Rep..

[102] Yeh K.-M., Kurup A., Siu L., Koh Y., Fung C.-P., Lin J.-C., Chen T.-L., Chang F.-Y., Koh T.-H. (2007). Capsular serotype K1 or K2, rather than magA and rmpA, is a major virulence determinant for *Klebsiella pneumoniae* liver abscess in Singapore and Taiwan. J. Clin. Microbiol..

[103] Fung C., Chang F., Lee S., Hu B., Kuo B.I., Liu C., Ho M., Siu L. (2002). A global emerging disease of *Klebsiella pneumoniae* liver abscess: Is serotype K1 an important factor for complicated endophthalmitis?. Gut.

[104] Athamna A., Ofek I., Keisari Y., Markowitz S., Dutton G., Sharon N. (1991). Lectinophagocytosis of encapsulated *Klebsiella pneumoniae* mediated by surface lectins of guinea pig alveolar macrophages and human monocyte-derived macrophages. Infect. Immun..

[105] Lee C.-H., Chuah S.-K., Chang C.-C., Chen F.-J. (2020). The surface protein fructose-1, 6 bisphosphate aldolase of *Klebsiella pneumoniae* serotype K1: Role of interaction with Neutrophils. Pathogens.

[106] Lee C.-H., Chang C.-C., Liu J.-W., Chen R.-F., Yang K.D. (2014). Sialic acid involved in hypermucoviscosity phenotype of *Klebsiella pneumoniae* and associated with resistance to neutrophil phagocytosis. Virulence.

[107] Opal S.M. (2014). Significance of sialic acid in *Klebsiella pneumoniae* K1 capsules. Virulence.

[108] Bullen J., Rogers H.J., Griffiths E. (1972). Iron binding proteins and infection. Br. J. Haematol..

[109] Carniel E. (2001). The Yersinia high-pathogenicity island: An iron-uptake island. Microbes Infect..

[110] Holden V.I., Bachman M.A. (2015). Diverging roles of bacterial siderophores during infection. Metallomics.

[111] Miethke M., Marahiel M.A. (2007). Siderophore-based iron acquisition and pathogen control. Microbiol. Mol. Biol. Rev..

[112] Holt K.E., Wertheim H., Zadoks R.N., Baker S., Whitehouse C.A., Dance D., Jenney A., Connor T.R., Hsu L.Y., Severin J. (2015). Genomic analysis of diversity, population structure, virulence, and antimicrobial resistance in *Klebsiella pneumoniae*, an urgent threat to public health. Proc. Natl. Acad. Sci. USA.

[113] Russo T.A., Gulick A.M. (2019). Aerobactin synthesis proteins as antivirulence targets in hypervirulent *Klebsiella pneumoniae*. ACS Infect. Dis..

[114] Zsila F., Beke-Somfai T. (2020). Human host-defense peptide LL-37 targets stealth siderophores. Biochem. Biophys. Res. Commun..

[115] Flo T.H., Smith K.D., Sato S., Rodriguez D.J., Holmes M.A., Strong R.K., Akira S., Aderem A. (2004). Lipocalin 2 mediates an innate immune response to bacterial infection by sequestrating iron. Nature.

[116] Bachman M.A., Lenio S., Schmidt L., Oyler J.E., Weiser J.N. (2012). Interaction of lipocalin 2, transferrin, and siderophores determines the replicative niche of *Klebsiella pneumoniae* during pneumonia. MBio.

[117] Shankar-Sinha S., Valencia G.A., Janes B.K., Rosenberg J.K., Whitfield C., Bender R.A., Standiford T.J., Younger J.G. (2004). The *Klebsiella pneumoniae* O antigen contributes to bacteremia and lethality during murine pneumonia. Infect. Immun..

[118] Hansen D.S., Mestre F., Albertí S.n., Hernández-Allés S., Álvarez D., Doménech-Sánchez A., Gil J., Merino S., Tomás J.M., Benedí V.J. (1999). *Klebsiella pneumoniae* lipopolysaccharide O typing: Revision of prototype strains and O-group distribution among clinical isolates from different sources and countries. J. Clin. Microbiol..

[119] Hsieh P.-F., Liu J.-Y., Pan Y.-J., Wu M.-C., Lin T.-L. (2013). Klebsiella pneumoniae peptidoglycan-associated lipoprotein and murein lipoprotein. J. Infect. Dis..

[120] Merino S., Camprubi S., Alberti S., Benedi V.-J., Tomas J. (1992). Mechanisms of *Klebsiella pneumoniae* resistance to complement-mediated killing. Infect. Immun..

[121] Schroll C., Barken K.B., Krogfelt K.A., Struve C. (2010). Role of type 1 and type 3 fimbriae in *Klebsiella pneumoniae* biofilm formation. BMC Microbiol..

[122] Wu C.-C., Huang Y.-J., Fung C.-P., Peng H.-L. (2010). Regulation of the *Klebsiella pneumoniae* Kpc fimbriae by the site-specific recombinase KpcI. Microbiology.

[123] Chou H.-C., Lee C.-Z., Ma L.-C., Fang C.-T., Chang S.-C., Wang J.-T. (2004). Isolation of a chromosomal region of *Klebsiella pneumoniae* associated with allantoin metabolism and liver infection. Infect. Immun..

[124] Cusa E., Obradors N., Baldomà L., Badía J., Aguilar J. (1999). Genetic analysis of a chromosomal region containing genes required for assimilation of allantoin nitrogen and linked glyoxylate metabolism in Escherichia coli. J. Bacteriol..

[125] Compain F., Babosan A., Brisse S., Genel N., Audo J., Ailloud F., Kassis-Chikhani N., Arlet G., Decré D. (2014). Multiplex PCR for detection of seven virulence factors and K1/K2 capsular serotypes of *Klebsiella pneumoniae*. J. Clin. Microbiol..

[126] Faïs T., Delmas J., Barnich N., Bonnet R., Dalmasso G. (2018). Colibactin: More than a new bacterial toxin. Toxins.

[127] Lan Y., Zhou M., Jian Z., Yan Q., Wang S., Liu W. (2019). Prevalence of pks gene cluster and characteristics of *Klebsiella pneumoniae*-induced bloodstream infections. J. Clin. Lab. Anal..

[128] Bulger J., MacDonald U., Olson R., Beanan J., Russo T.A. (2017). Metabolite transporter PEG344 is required for full virulence of hypervirulent *Klebsiella pneumoniae* strain hvKP1 after pulmonary but not subcutaneous challenge. Infect. Immun..

[129] Hao Z., Duan J., Liu L., Shen X., Yu J., Guo Y., Wang L., Yu F. (2020). Prevalence of community-acquired, hypervirulent *Klebsiella pneumoniae* isolates in Wenzhou, China. Microb. Drug Resist..

[130] Shankar C., Jacob J.J., Vasudevan K., Biswas R., Manesh A., Sethuvel D.P.M., Varughese S., Biswas I., Veeraraghavan B. (2020). Emergence of multidrug resistant hypervirulent ST23 *Klebsiella pneumoniae*: Multidrug resistant plasmid acquisition drives evolution. Front. Cell. Infect. Microbiol..

[131] Liu Y., Long D., Xiang T.-X., Du F.-L., Wei D.D., Wan L.-G., Deng Q., Cao X.-W., Zhang W. (2019). Whole genome assembly and functional portrait of hypervirulent extensively drug-resistant NDM-1 and KPC-2 co-producing *Klebsiella pneumoniae* of capsular serotype K2 and ST86. J. Antimicrob. Chemother..

[132] Liao W., Long D., Huang Q., Wei D., Liu X., Wan L., Feng Y., Zhang W., Liu Y. (2020). Rapid detection to differentiate hypervirulent *Klebsiella pneumoniae* (hvKp) from classical *K. pneumoniae* by identifying peg-344 with loop-mediated isothermal amplication (LAMP). Front. Microbiol..

[133] Asokan G.V., Ramadhan T., Ahmed E., Sanad H. (2019). WHO global priority pathogens list: A bibliometric analysis of medline-pubmed for knowledge mobilization to infection prevention and control practices in Bahrain. Oman Med. J..

[134] WHO (2024). WHO Bacterial Priority Pathogens List, 2024: Bacterial Pathogens of Public Health Importance, to Guide Research, Development, and Strategies to Prevent and Control Antimicrobial Resistance.

[135] Pendleton J.N., Gorman S.P., Gilmore B.F. (2013). Clinical relevance of the ESKAPE pathogens. Expert Rev Anti Infect Ther.

[136] Wyres K.L., Holt K.E. (2018). *Klebsiella pneumoniae* as a key trafficker of drug resistance genes from environmental to clinically important bacteria. Curr. Opin. Microbiol..

[137] Navon-Venezia S., Kondratyeva K., Carattoli A. (2017). *Klebsiella pneumoniae*: A major worldwide source and shuttle for antibiotic resistance. FEMS Microbiol. Rev..

[138] Lee C.-R., Lee J.H., Park K.S., Jeon J.H., Kim Y.B., Cha C.-J., Jeong B.C., Lee S.H. (2017). Antimicrobial resistance of hypervirulent *Klebsiella pneumoniae*: Epidemiology, hypervirulence-associated determinants, and resistance mechanisms. Front. Cell. Infect. Microbiol..

[139] Feng Y., Lu Y., Yao Z., Zong Z. (2018). Carbapenem-resistant hypervirulent *Klebsiella pneumoniae* of sequence type 36. Antimicrob. Agents Chemother..

[140] Zhang R., Lin D., Chan E.W.-c., Gu D., Chen G.-X., Chen S. (2016). Emergence of carbapenem-resistant serotype K1 hypervirulent *Klebsiella pneumoniae* strains in China. Antimicrob. Agents Chemother..

[141] Turton J.F., Payne Z., Coward A., Hopkins K.L., Turton J.A., Doumith M., Woodford N. (2018). Virulence genes in isolates of *Klebsiella pneumoniae* from the UK during 2016, including among carbapenemase gene-positive hypervirulent K1-ST23 and ‘non-hypervirulent’types ST147, ST15 and ST383. J. Med. Microbiol..

[142] Dong N., Yang X., Zhang R., Chan E.W.-C., Chen S. (2018). Tracking microevolution events among ST11 carbapenemase-producing hypervirulent *Klebsiella pneumoniae* outbreak strains. Emerg. Microbes Infect..

[143] El-Mokhtar M.A., Hetta H.F. (2018). Ambulance vehicles as a source of multidrug-resistant infections: A multicenter study in Assiut City, Egypt. Infect. Drug Resist..

[144] Xu M., Li A., Kong H., Zhang W., Chen H., Fu Y., Fu Y. (2018). Endogenous endophthalmitis caused by a multidrug-resistant hypervirulent *Klebsiella pneumoniae* strain belonging to a novel single locus variant of ST23: First case report in China. BMC Infect. Dis..

[145] Gurieva T., Dautzenberg M.J., Gniadkowski M., Derde L.P., Bonten M.J., Bootsma M.C. (2018). The transmissibility of antibiotic-resistant Enterobacteriaceae in intensive care units. Clin. Infect. Dis..

[146] Xie Y., Tian L., Li G., Qu H., Sun J., Liang W., Li X., Wang X., Deng Z., Liu J. (2018). Emergence of the third-generation cephalosporin-resistant hypervirulent *Klebsiella pneumoniae* due to the acquisition of a self-transferable bla DHA-1-carrying plasmid by an ST23 strain. Virulence.

[147] Cejas D., Fernández Canigia L., Rincón Cruz G., Elena A.X., Maldonado I., Gutkind G.O., Radice M.A. (2014). First isolate of KPC-2-producing Klebsiella pneumonaie sequence type 23 from the Americas. J. Clin. Microbiol..

[148] Zhang Y., Zeng J., Liu W., Zhao F., Hu Z., Zhao C., Wang Q., Wang X., Chen H., Li H. (2015). Emergence of a hypervirulent carbapenem-resistant *Klebsiella pneumoniae* isolate from clinical infections in China. J. Infect..

[149] Wei D.-d., Wan L.-G., Deng Q., Liu Y. (2016). Emergence of KPC-producing *Klebsiella pneumoniae* hypervirulent clone of capsular serotype K1 that belongs to sequence type 11 in Mainland China. Diagn. Microbiol. Infect. Dis..

[150] Huang Y.-H., Chou S.-H., Liang S.-W., Ni C.-E., Lin Y.-T., Huang Y.-W., Yang T.-C. (2018). Emergence of an XDR and carbapenemase-producing hypervirulent *Klebsiella pneumoniae* strain in Taiwan. J. Antimicrob. Chemother..

[151] Cannatelli A., Giani T., D'Andrea M.M., Di Pilato V., Arena F., Conte V., Tryfinopoulou K., Vatopoulos A., Rossolini G.M. (2014). MgrB inactivation is a common mechanism of colistin resistance in KPC-producing *Klebsiella pneumoniae* of clinical origin. Antimicrob. Agents Chemother..

[152] Hwang J.H., Handigund M., Hwang J.H., Cho Y.G., Kim D.S., Lee J. (2020). Clinical features and risk factors associated with 30-day mortality in patients with pneumonia caused by hypervirulent *Klebsiella pneumoniae* (hvKP). Ann. Lab Med..

[153] Asokan S., Jacob T., Jacob J., AlSosowaa A.A., Cherian T., Peijnenburg W.J.G.M., Vijayan S. (2025). *Klebsiella pneumoniae:* A growing threat in the era of antimicrobial resistance. Microbe.

[154] Bennett J., Dolin R., Blaser M. (2014). Mandell, Douglas, and Bennett’s Principles and Practice of Infectious Diseases: 2-Volume Set: Elsevier Health Sciences.

[155] Li J., Ren J., Wang W., Wang G., Gu G., Wu X., Wang Y., Huang M., Li J. (2018). Risk factors and clinical outcomes of hypervirulent *Klebsiella pneumoniae* induced bloodstream infections. Eur. J. Clin. Microbiol. Infect. Dis..

[156] Pomakova D., Hsiao C., Beanan J., Olson R., MacDonald U., Keynan Y., Russo T. (2012). Clinical and phenotypic differences between classic and hypervirulent Klebsiella pneumonia: An emerging and under-recognized pathogenic variant. Eur. J. Clin. Microbiol. Infect. Dis..

[157] Sobirk S.K., Struve C., Jacobsson S.G. (2010). Primary *Klebsiella pneumoniae* liver abscess with metastatic spread to lung and eye, a North-European case report of an emerging syndrome. Open Microbiol. J..

[158] Siu L.K., Yeh K.-M., Lin J.-C., Fung C.-P., Chang F.-Y. (2012). *Klebsiella pneumoniae* liver abscess: A new invasive syndrome. Lancet Infect. Dis..

[159] Lederman E.R., Crum N.F. (2005). Pyogenic liver abscess with a focus on *Klebsiella pneumoniae* as a primary pathogen: An emerging disease with unique clinical characteristics. Off. J. Am. Coll. Gastroenterol. | ACG.

[160] Pastagia M., Arumugam V. (2008). *Klebsiella pneumoniae* liver abscesses in a public hospital in Queens, New York. Travel Med. Infect. Dis..

[161] Chung D., Lee H., Park M., Jung S.-I., Chang H.-H., Kim Y.-S., Son J., Moon C., Kwon K., Ryu S. (2012). Fecal carriage of serotype K1 *Klebsiella pneumoniae* ST23 strains closely related to liver abscess isolates in Koreans living in Korea. Eur. J. Clin. Microbiol. Infect. Dis..

[162] Kao W.Y., Hwang C.Y., Chang Y.T., Su C.W., Hou M.C., Lin H.C., Lee F.Y., Lee S.D., Wu J.C. (2012). Cancer risk in patients with pyogenic liver abscess: A nationwide cohort study. Aliment. Pharmacol. Ther..

[163] Huang W.K., Chang J.C., See L.C., Tu H.T., Chen J.S., Liaw C.C., Lin Y.C., Yang T.S. (2012). Higher rate of colorectal cancer among patients with pyogenic liver abscess with *Klebsiella pneumoniae* than those without: An 11-year follow-up study. Color. Dis..

[164] Boltin D., Goldberg E., Bugaevsky O., Kelner E., Birkenfeld S., Gingold-Belfer R., Keller N., Niv Y., Dickman R. (2015). Colonic carriage of Streptococcus bovis and colorectal neoplasia: A prospective 17-year longitudinal case–control study. Eur. J. Gastroenterol. Hepatol..

[165] Srivastava I., Aldape M.J., Bryant A.E., Stevens D.L. (2017). Spontaneous C. ásepticum gas gangrene: A literature review. Anaerobe.

[166] Wu X., Liu J., Feng J., Shabbir M.A.B., Feng Y., Guo R., Zhou M., Hou S., Wang G., Hao H. (2022). Epidemiology, environmental risks, virulence, and resistance determinants of *Klebsiella pneumoniae* from dairy cows in Hubei, China. Front. Microbiol..

[167] Harada S., Doi Y. (2018). Hypervirulent *Klebsiella pneumoniae*: A call for consensus definition and international collaboration. J. Clin. Microbiol..

[168] Liu C., Shi J., Guo J. (2018). High prevalence of hypervirulent *Klebsiella pneumoniae* infection in the genetic background of elderly patients in two teaching hospitals in China. Infect. Drug Resist..

[169] Hetta H.F., Ramadan Y.N., Al-Kadmy I.M.S. (2025). Editorial for special issue “antibiotic combination therapy: A strategy to overcome bacterial resistance”. Biomedicines.

[170] Farhan S.M., Ibrahim R.A., Mahran K.M., Hetta H.F., Abd El-Baky R.M. (2019). Antimicrobial resistance pattern and molecular genetic distribution of metallo-β-lactamases producing Pseudomonas aeruginosa isolated from hospitals in Minia, Egypt. Infect. Drug Resist..

[171] de Oliveira Júnior N.G., Franco O.L. (2020). Promising strategies for future treatment of *Klebsiella pneumoniae* biofilms. Future Microbiol..

[172] Abid S.A., Al-Kadmy I.M.S., Aziz S.N., Garallah E.T., Aziz R.N., Ramadan Y.N., Hetta H.F. (2025). Bacterial dormancy: Strategies and molecular mechanisms for a sleeping beauty system. Rev. Res. Med. Microbiol..

[173] Tan Y.-M., Chung A.Y.-F., Chow P.K.-H., Cheow P.-C., Wong W.-K., Ooi L.L., Soo K.-C. (2005). An appraisal of surgical and percutaneous drainage for pyogenic liver abscesses larger than 5 cm. Ann. Surg..

[174] Wang Z., Cai R., Wang G., Guo Z., Liu X., Guan Y., Ji Y., Zhang H., Xi H., Zhao R. (2021). Combination therapy of phage vB_KpnM_P-KP2 and gentamicin combats acute pneumonia caused by K47 serotype *Klebsiella pneumoniae*. Front. Microbiol..

[175] Hetta H.F., Rashed Z.I., Ramadan Y.N., Al-Kadmy I.M.S., Kassem S.M., Ata H.S., Nageeb W.M. (2023). Phage therapy, a salvage treatment for multidrug-resistant bacteria causing infective endocarditis. Biomedicines.

[176] Zurabov F., Zhilenkov E. (2021). Characterization of four virulent *Klebsiella pneumoniae* bacteriophages, and evaluation of their potential use in complex phage preparation. Virol. J..

[177] Feldman M.F., Bridwell A.E.M., Scott N.E., Vinogradov E., McKee S.R., Chavez S.M., Twentyman J., Stallings C.L., Rosen D.A., Harding C.M. (2019). A promising bioconjugate vaccine against hypervirulent *Klebsiella pneumoniae*. Proc. Natl. Acad. Sci. USA.

[178] Zargaran F.N., Akya A., Rezaeian S., Ghadiri K., Lorestani R.C., Madanchi H., Safaei S., Rostamian M. (2021). B cell epitopes of four fimbriae antigens of *Klebsiella pneumoniae*: A comprehensive in silico study for vaccine development. Int. J. Pept. Res. Ther..

[179] Diago-Navarro E., Calatayud-Baselga I., Sun D., Khairallah C., Mann I., Ulacia-Hernando A., Sheridan B., Shi M., Fries B.C. (2017). Antibody-based immunotherapy to treat and prevent infection with hypervirulent *Klebsiella pneumoniae*. Clin. Vaccine Immunol..

[180] Hetta H.F., Al-Kadmy I.M., Khazaal S.S., Abbas S., Suhail A., El-Mokhtar M.A., Ellah N.H.A., Ahmed E.A., Abd-Ellatief R.B., El-Masry E.A. (2021). Antibiofilm and antivirulence potential of silver nanoparticles against multidrug-resistant Acinetobacter baumannii. Sci. Rep..

[181] Hetta H.F., Ramadan Y.N., Rashed Z.I., Alharbi A.A., Alsharef S., Alkindy T.T., Alkhamali A., Albalawi A.S., Battah B., Donadu M.G. (2024). Quorum sensing inhibitors: An alternative strategy to win the battle against multidrug-resistant (MDR) bacteria. Molecules.

[182] Hetta H.F., Sirag N., Alsharif S.M., Alharbi A.A., Alkindy T.T., Alkhamali A., Albalawi A.S., Ramadan Y.N., Rashed Z.I., Alanazi F.E. (2024). Antimicrobial peptides: The game-changer in the epic battle against multidrug-resistant bacteria. Pharmaceuticals.

[183] Hetta H.F., Ramadan Y.N., Al-Harbi A.I., Ahmed E.A., Battah B., Abd Ellah N.H., Zanetti S., Donadu M.G. (2023). Nanotechnology as a promising approach to combat multidrug resistant bacteria: A comprehensive review and future perspectives. Biomedicines.

[184] Abo-Shama U.H., El-Gendy H., Mousa W.S., Hamouda R.A., Yousuf W.E., Hetta H.F., Abdeen E.E. (2020). Synergistic and antagonistic effects of metal nanoparticles in combination with antibiotics against some reference strains of pathogenic microorganisms. Infect. Drug Resist..

